# Spatial Regulation of Endocytosis and Adhesion Formation Governs Breast Cancer Cell Migration Under Confinement

**DOI:** 10.3390/bioengineering12111148

**Published:** 2025-10-23

**Authors:** Emily T. Chan, Travis H. Jones, Cristopher M. Thompson, Hariharan Kannan, Malcolm W. D’Souza, Mushtaq M. Ali, Cömert Kural, Jonathan W. Song

**Affiliations:** 1 Interdisciplinary Biophysics Graduate Program, The Ohio State University, Columbus, OH 43210, USA; 2 Department of Mechanical and Aerospace Engineering, The Ohio State University, Columbus, OH 43210, USA; jones.3318@osu.edu; 3 Department of Physics, The Ohio State University, Columbus, OH 43210, USA; thompson.3962@buckeyemail.osu.edu; 4 Department of Chemistry and Biochemistry, The Ohio State University, Columbus, OH 43210, USA; kannan.84@buckeyemail.osu.edu; 5 Biomedical Science, College of Medicine, The Ohio State University, Columbus, OH 43210, USA; dsouza.110@buckeyemail.osu.edu; 6 Biological Systems Engineering, College of Agricultural and Life Sciences, University of Wisconsin—Madison, Madison, WI 53706, USA; mmali5@wisc.edu; 7 Comprehensive Cancer Center, The Ohio State University, Columbus, OH 43210, USA

**Keywords:** biophysics, microfluidics, mechanobiology, chemokine gradient, receptor dynamics

## Abstract

Cell migration through confined spaces is a critical step in cancer metastasis, yet the spatial regulation of endocytosis and adhesion dynamics during this process remains poorly understood. To investigate this, we adapted a microfluidic platform that generates stable, spatially linear biochemical gradients across 5 μm-tall migration channels. COMSOL simulations and optical calibration using FITC-dextran confirmed that gradients form reliably within 5 min. The microdevice also supports long-term live imaging and is compatible with both spinning disk confocal and total internal reflection fluorescence structured illumination microscopy modalities, enabling high-resolution visualization of adhesion and endocytic structures. By leveraging this platform for spatially restricted drug delivery, we locally applied the endocytic inhibitor Dyngo-4a to either the front or rear of migrating cells. This revealed that front-targeted endocytic inhibition preserved or increased leading-edge enrichment of paxillin and the clathrin adaptor AP-2, whereas rear-targeted inhibition eliminated paxillin polarity and reduced AP-2 polarity. These changes were accompanied by a significant increase in cell migration speed under front-targeted inhibition, while rear-targeted inhibition had no significant effect on speed and neither treatment altered persistence. Together, these findings suggest that endocytic polarity regulates adhesion dynamics and cell migration under confinement, offering a mechanistic insight into processes relevant to cancer cell invasion.

## 1. Introduction

Cell migration is central to numerous physiological processes, including immune surveillance [1], wound healing [2], and embryonic development [3]. In cancer, however, similar migratory machinery is co-opted to drive tumor invasion and metastasis [2,4], requiring cells to traverse narrow, confined spaces within the extracellular matrix (ECM) [2,4,5]. These spatial constraints dramatically alter cell morphology, cytoskeletal architecture, and intracellular signaling [5,6,7]. Despite the biological and clinical importance of confined cell migration, how cells integrate spatial biochemical cues with physical confinement to coordinate motility remains incompletely understood.

A key but underexplored component of this integration is the spatial regulation of membrane trafficking processes such as endocytosis. Clathrin-mediated endocytosis (CME) orchestrates key events during cell migration, including receptor internalization and focal adhesion (FA) turnover [8,9,10]. Prior studies show that CME and FAs exhibit spatial asymmetries: CME is reduced at the leading edge of migrating cells, while mature FAs preferentially accumulate toward the rear [11,12]. However, it remains unclear how these polarized distributions are maintained or altered under physical confinement. Most conventional systems lack the spatial resolution and environmental control to probe these dynamics at subcellular resolution in live migrating cells.

Dissecting the region-specific roles of endocytosis in migration requires spatially targeted, rather than global, perturbations. Endocytosis is inherently polarized in migrating cells, with distinct roles at the front and rear [13,14,15]. Microfluidic systems that combine chemotactic gradients with cell-sized channels have been developed to study confined migration and have proven compatible with high-resolution microscopy [4,16,17]. These platforms can establish stable gradients, minimize shear stress, and support live-cell imaging, but their use has largely focused on migration mechanics and chemotaxis rather than localized regulation of endocytosis.

Previous studies using pharmacological inhibition or genetic suppression of dynamin, a large GTPase required for membrane fission during CME, have examined the effects of globally reducing endocytosis on cell migration [18,19]. However, because these approaches act uniformly across the cell, they were unable to resolve region-specific functions of endocytosis at the leading and trailing edges. To address this limitation, we developed a microfluidic platform that enables spatially restricted inhibition of CME to either the front or rear of migrating cells, allowing us to distinguish how localized endocytic activity regulates adhesion dynamics, polarity, and migration behavior under confinement. The platform generates reproducible chemotactic gradients within narrow, cell-sized channels that are optically transparent for confocal and super-resolution microscopy. Using this system, we show that localized endocytic inhibition produces distinct directional effects on adhesion distribution, polarity maintenance, and migration speed, underscoring the importance of spatial membrane dynamics in confined cell migration and providing mechanistic insight into how endocytic and adhesive asymmetries enable cancer cell invasion.

## 2. Methods

### 2.1. Cell Lines and Reagents

Genome-edited AP2-eGFP expressing human breast cancer SUM159 cell lines (eGFP incorporated at the C-terminus of the σ2 subunit of AP-2, a marker for endocytic clathrin structures) were kindly gifted by Dr. Tomas Kirchhausen (Harvard Medical School). SUM159 cells were cultured in complete media consisting of phenol red-free Ham’s F-12 (Caisson Labs, Smithfield, UT, USA, HFL05), supplemented with 5% fetal bovine serum (Thermo Fisher Scientific Inc., Waltham, MA, USA, A5256701), 1% penicillin and streptomycin (Thermo Fisher Scientific Inc., Waltham, MA, USA, 15140122), 1 μg/mL hydrocortisone (Sigma-Aldrich, St. Louis, MO, USA, H-4001), 5 μg/mL insulin (Cell Applications Inc., San Diego, CA, USA, 128-100), and 10 mM 4-(2-hydroxyethyl)-1- piperazine-ethane-sulfonic acid (HEPES, Corning Inc., Corning, NY, USA, 25-060-CI) at pH 7.4. All cells were maintained at 37 °C and 5% CO_2_ and passaged every 2–3 days. Cell stock solutions were replaced after a maximum of 8 passages.

### 2.2. Fabrication of Microfluidic Migration Devices

Microfluidic migration devices were fabricated using standard soft lithography techniques adapted from prior designs that generate stable, linear gradients across cell-sized channels [16,20]. The device features two chambers (100 μm tall, 100 μm wide, 1400 μm long) connected by 30 parallel migration channels (5 μm tall, 20 μm wide, 150 μm long) with two inlets and one outlet. Additional fabrication details and validation of device dimensions by scanning electron microscopy are provided in Supplementary Methods S1 and S2. Multi-level SU-8 photoresist layers (Kayaku Advanced Materials, Inc., Westborough, MA, USA) were patterned on silicon wafers to form the master mold. Polydimethylsiloxane (PDMS, Dow Corning Corporation, Midland, MI, USA, Sylgard 184 Silicone Elastomer) replicas were cast at a 10:1 base-to-crosslinker ratio, cured at 65 °C for 2 h, and bonded to a No. 1.5 glass coverslip inside a 35 mm dish (MatTek, Ashland, MA, USA, P35G-1.5-20-C) using plasma oxidation (30 W, 650 mTorr, 70 s; Harrick Plasma, Ithaca, NY, USA, PDC-001). After bonding, devices were baked at 65 °C for 10 min to ensure permanent sealing.

### 2.3. Gradient Characterization in Migration Devices

Gradients were validated both computationally and experimentally. COMSOL Multiphysics simulations (CFD + Transport of Diluted Species modules, COMSOL Multiphysics v6.2) were used to simulate fluid flow and molecular diffusion within the migration device geometry reconstructed from measured dimensions. Boundary conditions included no-slip walls, equal inlet pressures, and a constant outlet withdrawal rate of 5 μL/h to maintain low-shear conditions representative of cell migration assays.

For experimental validation, a 10 kDa FITC-dextran (1 mg/mL, Sigma-Aldrich, St. Louis, MO, USA, SLCF8359) was introduced into one inlet of the microfluidic device, while 1X PBS (Thermo Fisher Scientific Inc., Waltham, MA, USA, 10010023) was introduced to the opposite inlet to generate a diffusion-based gradient. The outlet was connected to a syringe pump (Harvard Apparatus, Holliston, MA, USA, 70-3007) operating in withdrawal mode at 5 μL/h to ensure steady flow. Fluorescence imaging was performed on a spinning-disk confocal microscope (20X objective, 488 nm excitation; see *Spinning disk confocal image acquisition*) maintained at 37 °C. Intensity profiles along the migration channels were extracted and normalized in FIJI v2.14.0/1.54f [21] for subsequent gradient quantification. Full details of the COMSOL model geometry, mesh parameters, and experimental calibration using FITC-dextran are provided in Supplementary Methods S3 and S4.

### 2.4. Cell Loading into Migration Device

Devices bonded to No. 1.5 glass coverslips were sterilized by UV exposure and coated with 100 μg/mL fibronectin (Sigma-Aldrich, St. Louis, MO, USA, FC010) for 1.5 h at 37 °C and 5% CO_2_ to promote cell adhesion. SUM159 cells were harvested with 0.05% trypsin-EDTA (Thermo Fisher Scientific Inc., Waltham, MA, USA, 25200056), resuspended in phenol red-free F-12 complete media (1 × 10^5^ cells/mL), and introduced into the device chambers by passive flow. After cell attachment, the devices were washed and incubated in serum-free media for 1.5 h under a withdrawal rate of 5 μL/h to allow stabilization before migration assays. Full details of the cell loading and equilibration procedures are provided in Supplementary Method S5.

### 2.5. Acquisition of Cell Migration Speeds and Persistence

Following serum starvation, time-lapse imaging of single-cell migration was performed using a dual phase/fluorescence LED microscope (Leica Microsystems GmbH, Wetzlar, Germany, PAULA). Epidermal growth factor (EGF, Thermo Fisher Scientific Inc., Waltham, MA, USA, AF-100-15-500UG, 20 ng/mL) was introduced into one inlet of the device to establish a chemotactic gradient. For inhibition experiments, 5 μM Dyngo-4a (Abcam, Cambridge, UK, ab120689) was applied either to the same inlet as EGF (front treatment) or to the opposite inlet (rear treatment). Cells were imaged every 10 min for up to 5 h at 37 °C and 5% CO_2_. Detailed loading volumes, timing, and gradient equilibration procedures are provided in Supplementary Method S6.

### 2.6. Analysis of Cell Migration Speeds and Persistence

Cell migration experiments were conducted in at least three independent replicates, with 8–10 cells analyzed per condition. Time-lapse images of cell motility were analyzed over a 2–4-h period using the FIJI plugin MTrackJ [22] (https://imagescience.org/meijering/software/mtrackj/, accessed on 14 July 2024). For all conditions, migration was analyzed after a 2-h incubation period to allow Dyngo-4a treatment to take effect and to ensure flow stabilization. Cell nuclei were tracked frame-by-frame to calculate displacement and velocity, beginning when each nucleus entered the microchannel and ending upon exit or completion of the time-lapse. Cells undergoing division or obstructed by another cell in either direction of the gradient were excluded. Cell persistence was defined as net displacement divided by total path length, and differences among conditions were assessed using two-way ANOVA (*p*-value < 0.05 was considered statistically significant, see *Statistics and reproducibility*).

### 2.7. Immunofluorescence Imaging of Migrated Cells

Immediately after migration assays, devices were removed from the incubator and cells were fixed with 3.7% paraformaldehyde (Sigma-Aldrich, St. Louis, MO, USA, P6148) for 15 min at room temperature. After fixation, cells were washed with PBS (Thermo Fisher Scientific Inc., Waltham, MA, USA, 10010023), permeabilized with 0.1% Triton X-100 (Sigma-Aldrich, St. Louis, MO, USA, T8787) for 15 min, and blocked overnight at 4 °C in PBS containing 2% rat serum (Thermo Fisher Scientific Inc., Waltham, MA, USA, 10-710-C) and 0.1% Tween-20 (Sigma-Aldrich, St. Louis, MO, USA, P9416). The following day, samples were incubated for 1 h with paxillin primary antibody (Thermo Fisher Scientific Inc., Waltham, MA, USA, AHO0492; 1:500 dilution) and then for 45 min with fluorochrome-conjugated secondary antibody (Thermo Fisher Scientific Inc., Waltham, MA, USA, A-21124; 1:3000 dilution). Nuclei were stained with Hoechst dye (Thermo Fisher Scientific Inc., Waltham, MA, USA, H21486; 1:2000 dilution) before imaging.

Imaging was performed using a spinning-disk confocal microscope (see *Spinning disk confocal image acquisition*) equipped with a 100X/1.45 NA objective (Nikon Corporation, Tokyo, Japan) and 405, 488, and 561 nm excitation. Z-stacks were acquired at 0.5 µm intervals (11–13 planes) to capture AP-2 and paxillin distributions throughout the cell volume. The Nikon Perfect Focus System maintained focus at the glass-PDMS interface, and an additional plane 1 µm below the focal position was included to capture the basal membrane. Cells were selected for imaging if they were fully confined within a single channel, non-dividing, and unobstructed by neighboring cells.

### 2.8. Spinning Disk Confocal Image Acquisition

High-resolution imaging of AP-2 and paxillin was performed using a Nikon Ti-E fluorescence microscope equipped with a CSU-W1 spinning disk unit (Yokogawa Electric Corporation, Tokyo, Japan), a 100X Plan-Apochromat Lambda objective (1.45 NA, Nikon Corporation, Tokyo, Japan), an sCMOS camera (Prime 95B; Teledyne Photometrics, Tucson, AZ, USA), and 405 nm, 488 nm, and 561 nm excitation lasers. Three-dimensional images of AP-2, paxillin, and cell nuclei were acquired using NIS Elements software v4.11.0.

### 2.9. Analysis of Focal Adhesions and Clathrin Pits

Confocal z-stacks were processed for quantitative analysis of paxillin and AP-2-labeled endocytic structures in FIJI [21]. After background subtraction, images were converted to 8-bit and projected as maximum-intensity images. Cell boundaries were identified using the Balloon Segmentation plugin (https://imagej.net/plugins/balloon, accessed on 20 October 2024), and a custom FIJI macro divided each cell into front and rear halves of equal cell area. These regions of interest (ROIs) were used to quantify front-to-rear differences in protein distributions.

Fluorescence signals were segmented using the Moments thresholding method. For AP-2, a watershed step was added to separate overlapping puncta, whereas paxillin was analyzed directly due to its diffuse appearance. AP-2 particle areas were measured within the front and rear ROIs, and paxillin and AP-2 intensities were normalized to total-cell fluorescence to obtain relative front-to-rear ratios.

### 2.10. Total Internal Reflection Fluorescence Structured Illumination Microscope (TIRF-SIM) Image Acquisition

TIRF-SIM images were acquired using a custom-built system previously described [23]. This system is constructed on an inverted Nikon Eclipse TI-E microscope body (Nikon Corporation, Tokyo, Japan), equipped with an Olympus APO 100 × 1.49 NA objective (Olympus Corporation, Tokyo, Japan) and a Hamamatsu ORCA-Fusion BT sCMOS camera (Hamamatsu Photonics K.K., Hamamatsu City, Shizuoka, Japan). Structured laser illumination (488 nm or 561 nm, 300 mW; Coherent Corp., Saxonburg, PA, USA, SAPPHIRE LP) is produced via an acousto-optic tunable filter (AOTF; AA Quanta Tech, Orsay, France, AOTFnC-400.650-TN), a polarizing beam splitter, an achromatic half-wave plate (HWP; Bolder Vision Optik, Boulder, CO, USA, BVO AHWP3), and a ferroelectric spatial light modulator (SLM; Forth Dimension Displays Ltd., Dalgety Bay, Fife, UK, QXGA-3DM-STR). Images acquired with varying phases and orientations are subsequently reconstructed into a super-resolution image using the HiFi-SIM algorithm [24].

### 2.11. Statistics and Reproducibility

All data presented reflect biologically independent experiments, with most conditions repeated at least three times. Cases with only two replicates are indicated in the figure legends. Statistical analyses and data visualization were performed using RStudio v2023.06.1, GraphPad Prism 10, and Microsoft Excel v16.102.1.

Unless otherwise noted, statistical tests were performed on experimental means (N), with individual cells or particles (n) treated as technical replicates and displayed in plots to illustrate variability [25]. For polarity analyses, log_2_(leading edge/trailing edge) fluorescence intensity ratios were calculated per cell. Group means were compared against zero (corresponding to equal leading and trailing intensities) to assess polarity within each condition, and across conditions (Control, Dyngo-4a front, Dyngo-4a rear) using one-way ANOVA with multiple-comparisons. Cumulative probability plots of log_2_-transformed AP-2 particle sizes were generated from pooled particles, with statistical comparisons performed on experiment-level means using Dunn’s multiple comparisons test. Migration speed and persistence were compared across conditions using one-way ANOVA on experimental means. *p*-values are reported as follows: *p* > 0.05, *: *p* ≤ 0.05, **: *p* ≤ 0.01, ***: *p* ≤ 0.001, ****: *p* ≤ 0.0001.

## 3. Results 

### 3.1. A Stable EGF Gradient Exists Across All Microchannels

**Figure 1:**
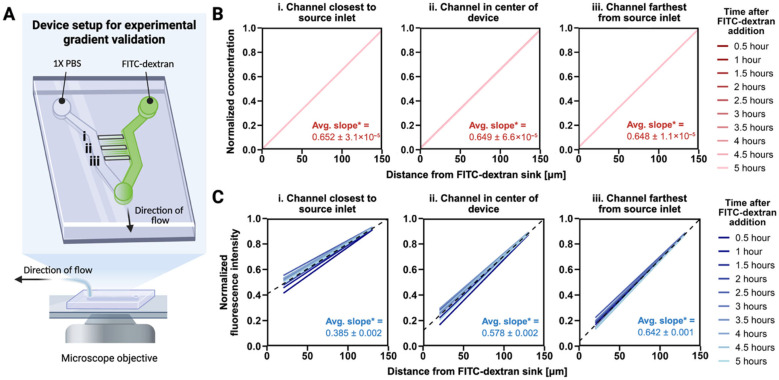
**Validation of stable FITC-dextran gradients across microfluidic migration channels.** (**A**) Experimental setup illustrating 10 kDa FITC-dextran introduced through the source inlet and sampled at defined channels across the device (i: closest to the source inlet, ii: center, iii: farthest from the source inlet). Flow is maintained using a syringe pump at a low flow rate, and the microscope objective is positioned under the migration channels. (**B**) Simulated gradients showing representative normalized concentration profiles at three channel positions, plotted against distance from the FITC-dextran sink. * Slopes are scaled by a factor of 100 for readability. (**C**) Experimental gradients showing normalized fluorescence intensity of FITC-dextran plotted against distance from the sink at three channel positions (N = 3). Data are truncated at the channel entrance and exit due to high out-of-plane fluorescence. The dashed black line represents the average linear fit across all timepoints. * Slopes are scaled by a factor of 100 for readability.

We designed and fabricated our platform to generate spatially linear biochemical gradients across confined, parallel microchannels while minimizing flow-mediated mechanical stimulation (Figure 1A) [16]. At a low flow rate of 5 µL/h, COMSOL Multiphysics v6.7 simulations confirmed that fluid flow remained restricted to the chambers of the device, with negligible velocity in the migration channels (Figure S1A). A z-profile of velocity magnitude across all 30 channels demonstrated that flow at the channel midplane was nearly zero (Figure S1B,C). We estimated shear stress in the chamber regions analytically (Hagen-Poiseuille relation for rectangular ducts) and confirmed using a COMSOL creeping-flow simulation, which yielded values on the order of 0.07 dyn/cm^2^. This is well below the threshold reported to induce migration through flow-dependent mechanisms, indicating that cell migration in this system is governed by the imposed biochemical gradient rather than shear forces [26].

To characterize the physical structure of the platform, we used scanning electron microscopy to confirm key microdevice dimensions, including a channel height of ~5 µm, width of 20 µm, and the spacing profile between adjacent channels (Figure S2A). We then performed optical calibration using 10 kDa FITC-dextran, selected for its comparable molecular weight to epidermal growth factor (EGF, ~6 kDa), and observed a linear relationship between fluorescence intensity and concentration (Figure S2B), enabling precise quantification of gradient strength using fluorescence imaging. COMSOL simulations predicted that a steady-state gradient would form within 3 min, with consistent and steep linear concentration profiles across representative channels (Figure 1B and Figure S1D and Movie S1). Time-lapse imaging confirmed that experimental gradients formed within 5 min and remained stable throughout the 5 h imaging period used for analyzing cell migration and paxillin/AP-2 formation, although the resulting slopes were shallower than those predicted by simulation (Figure 1C and Figure S2C). Unexpectedly, the shallowest gradients were observed in channels closest to the source inlet rather than those furthest away. This discrepancy is unlikely to result from time-dependent mixing delays alone, and more plausibly reflects subtle asymmetries in device geometry or residual hydrodynamic flow between channels that were not captured in the simulation. Despite these differences, the device reliably produced spatially stable gradients across all channels. For downstream migration analyses, we focused on cells in the middle to bottom 15 channels of the microdevice, where experimental gradients most closely aligned with model predictions.

### 3.2. Microdevice Design Supports High-Resolution Imaging of Subcellular Structures During Directed Cell Migration

**Figure 2:**
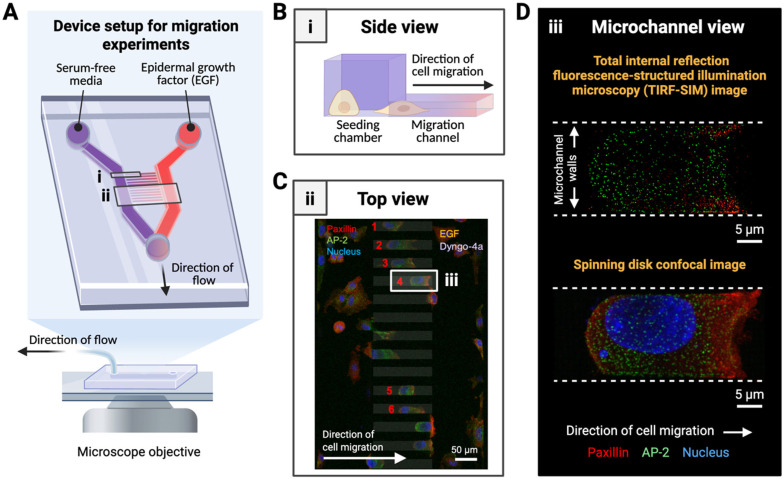
**Device design and imaging modalities for characterizing cancer cells during migration.** (**A**) Experimental setup of the microfluidic device used for migration assays, showing the introduction of EGF through the source inlet and the microscope objective positioned beneath the migration channels. Two representative regions are indicated by (i) and (ii), corresponding to panels (**B**,**C**), respectively. (**B**) Side view of the microchannel structure, highlighting the height difference between the seeding chamber and the 5 μm-tall migration channels. (**C**) Maximum intensity projection showing a top view of a portion of the device in an experiment where Dyngo-4a was applied to the same chamber as EGF. Cells endogenously express AP2-eGFP (green) and are immunostained for paxillin-mCherry (red) and Hoechst for the nucleus (blue). Tracked cells (6 cells) are indicated by red numbers. Arrow indicates the direction of cell migration. A representative region indicated by (iii) corresponds to panel (**D**). Scale bar: 50 μm. (**D**) Maximum intensity projections of representative migrating cells acquired using TIRF-SIM (top) and spinning disk confocal microscopy (bottom). Cells are stained for paxillin (red) and the nucleus (blue), and endogenously express AP2-eGFP (green). Dashed lines indicate the boundaries of the migration channel walls. Arrow indicates direction of cell migration. Scale bar: 5 μm.

A key attribute of our migration platform is its compatibility with high-resolution and super-resolution microscopy, enabling direct visualization of subcellular structures during chemotactic migration. We engineered the microchannel heights to be 5 μm, which both constrains cell morphology for axial alignment and permits optical access from below the device using high numerical aperture objectives (Figure 2A,B and Figure S3). This allowed us to confirm that cells migrating through the channels remain within the working distance of oil-immersion lenses used in high resolution spinning disk confocal and total internal reflection fluorescence structured illumination microscopy (TIRF-SIM).

We tested this compatibility by imaging migrating cells expressing endogenous AP2-eGFP (a marker for CME structures), immunostained for paxillin-mCherry (a focal adhesion marker), and dyed with nuclear Hoechst (Figure 2C and Figure S4A). Both TIRF-SIM and spinning disk confocal modalities produced high-resolution images within the channel confines, resolving distinct spatial distributions of adhesion and endocytic proteins (Figure 2D and Figure S4B,C, Movies S2 and S3). In cells located outside the channels, AP-2 and paxillin displayed more uniform distributions, and polarization was less apparent (Figure S4B,C). These observations underscore how microchannel confinement and chemotactic cues enhance spatial polarization of adhesion and endocytic machinery. Since we aim to visualize adhesive and endocytic structures throughout the full depth of the cell, for the remainder of the text, we focus on cells imaged in 3D using spinning disk confocal microscopy.

### 3.3. Front–Rear Endocytic Inhibition Reveals Spatial Control of Paxillin and AP-2 Distribution

To examine how endocytic activity influences adhesion dynamics during directed migration, we applied Dyngo-4a, a small molecule inhibitor of dynamin-mediated CME [18,27,28], to either the front or rear chamber of the microfluidic device. This spatially selective delivery approach allowed us to perturb specific regions of the cell without altering the entire pericellular microenvironment.

**Figure 3:**
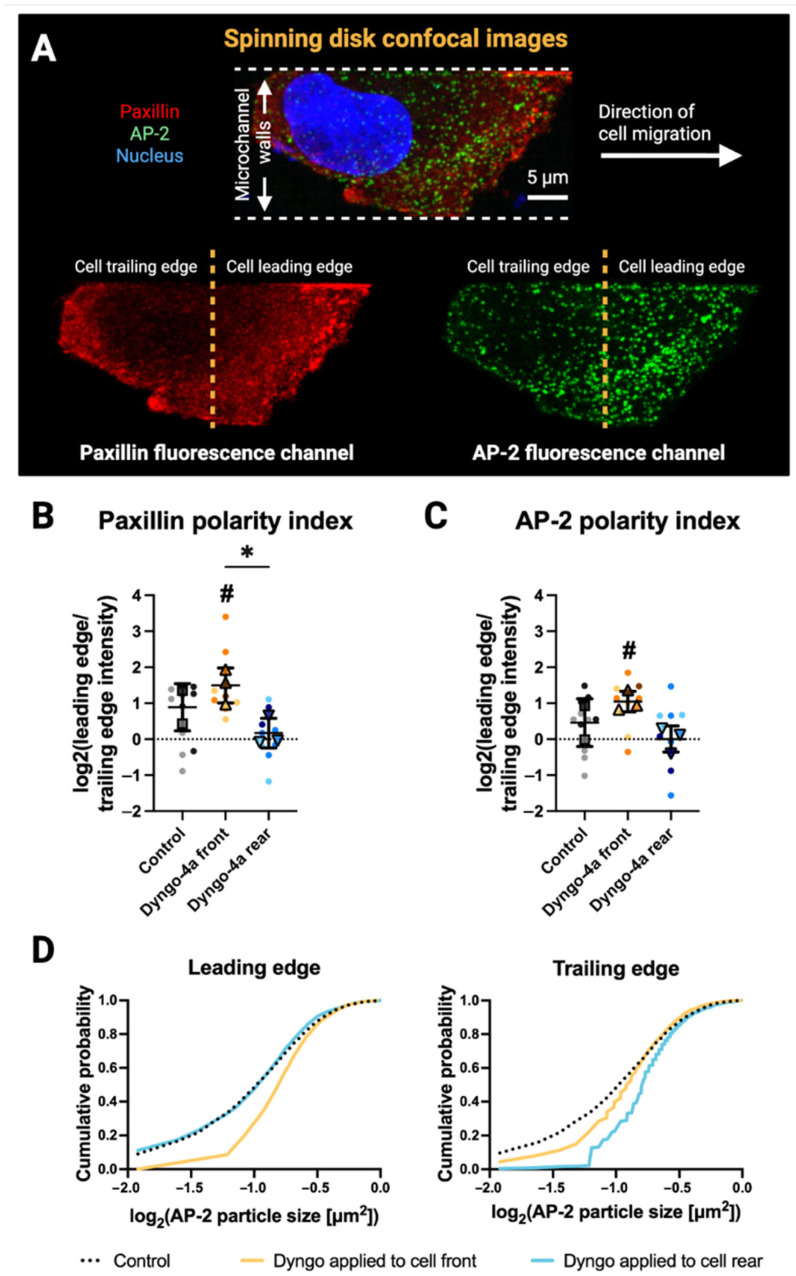
**Inhibition of endocytosis alters the front–rear distribution of paxillin and AP-2 structures, as well as the size of AP-2 particles.** (**A**) Maximum intensity projection of spinning disk confocal images showing a migrating cell within a microchannel, displaying paxillin (red), AP-2 (green), and the nucleus (blue). White dashed lines indicate the microchannel walls; yellow dashed lines denote the division between the cell leading and trailing edges. Arrow indicates direction of cell migration. Scale bar: 5 μm. (**B**) Quantification of log_2_(leading edge/trailing edge) fluorescence intensity ratios for paxillin under control conditions and with Dyngo-4a applied to the front or rear of the cell. Individual cells are shown as dots, color-coded by independent experiments (Control, N = 2; gray; Dyngo-4a front, N = 3, yellow; Dyngo-4a rear, N = 3, blue). Larger overlay points (squares = Control, upward triangles = Dyngo-4a front, downward triangles = Dyngo-4a rear) represent the mean from each experiment, which were used for statistical comparisons; error bars indicate mean ± standard deviation across experiments. Sample sizes: Control (n = 12), Dyngo-4a front (n = 9), Dyngo-4a rear (n = 9). Statistical significance between groups is shown with asterisks (* *p* < 0.05), while significance relative to zero (no front–rear difference) is indicated with #. (**C**) Quantification of log_2_(leading edge/trailing edge) AP-2 fluorescence intensity under the same conditions as (**B**). (**D**) Cumulative distribution plots of log_2_-transformed AP-2 particle sizes at the leading and trailing edges. Data represent pooled particles from three independent experiments (N = 3), with statistical comparisons performed on experiment means. All pairwise comparisons are significant (*p* < 0.001), except for Control vs. Dyngo-4a applied to the rear at the leading edge, and Control vs. Dyngo-4a applied to the front at the trailing edge.

Following gradient exposure and cell migration, we fixed and immunostained endogenously expressing AP2-eGFP cells for paxillin and nuclei (Figure 3A and Figure S5 and Movie S3), then analyzed them using a custom front–rear segmentation pipeline (Figure S6). High-resolution imaging revealed that inhibiting endocytosis at either end of the cell disrupted the polarized distribution of paxillin and AP-2 fluorescence (Figure 3B,C). Quantification of log_2_(leading edge/trailing edge) fluorescence intensity ratios showed that paxillin was significantly front-enriched under front-targeted Dyngo-4a treatment (*p* = 0.034, indicated by #) (Figure 3B). In contrast, rear-targeted inhibition eliminated this front–rear asymmetry/polarity, with ratios not significantly different from zero (*p* = 0.547) and significantly lower than front-targeted treatment (*p* = 0.047, indicated by *). Similarly, AP-2 was front-enriched under front-targeted Dyngo-4a treatment (*p* = 0.024, indicated by #), whereas rear-targeted inhibition showed no detectable polarity (*p* = 0.984) and yielded lower ratios than front-targeted treatment (*p* = 0.0613), although this difference did not reach significance (Figure 3C). This pattern suggests that rear-endocytosis plays a particularly important role in sustaining front–rear polarity of paxillin and AP-2, while front-endocytosis is less critical and may even promote stronger leading-edge enrichment.

Particle-level analysis of AP-2 puncta further showed that Dyngo-4a treatment altered particle size distributions (Figure 3D). Cumulative probability plots of log_2_-transformed particle sizes revealed a rightward shift at the leading edge under front-targeted Dyngo-4a treatment (*p* < 0.0001), whereas rear-targeted Dyngo-4a treatment showed no significant difference from control (*p* = 0.988). At the trailing edge, rear-targeted Dyngo-4a treatment resulted in a significant rightward shift (*p* < 0.0001), while front-targeted treatment was not significantly different from control (*p* = 0.7202), consistent with the accumulation of larger endocytic structures. This pattern reflects the mechanism of Dyngo-4a, which blocks scission of clathrin-coated pits but not their initiation or growth [18,29], resulting in stalled and enlarged endocytic intermediates. Together, these localized changes indicate that active endocytosis is required to maintain the polarized adhesion and endocytic distributions during cell migration.

### 3.4. Endocytic Inhibition Differentially Impacts Migration Speed and Persistence Depending on the Site of Application

**Figure 4:**
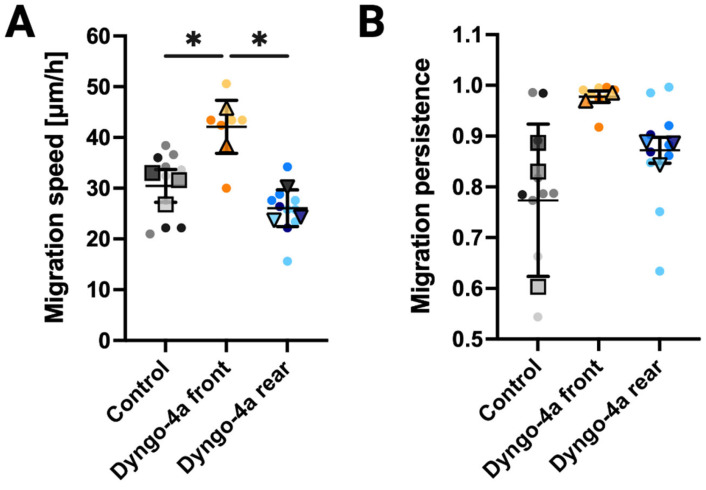
**Inhibition of endocytosis alters migration speed and persistence in single cells.** (**A**) Migration speed of cells under control conditions, or with Dyngo-4a applied to the cell front or rear. (**B**) Migration persistence of cells under the same conditions as in (**A**). Persistence is defined as the ratio of net displacement to total path length, with values closer to 1 indicating more directed migration. In our 1D microchannel geometry, this metric captures reversals in migration direction along the channel axis. Single-cell migration trajectories are shown as dots, color-coded by independent experiments (Control, N = 3, gray; Dyngo-4a applied to cell front, N = 2, yellow; Dyngo-4a applied to cell rear, N = 3, blue). Larger overlay points (squares = Control, upward triangles = Dyngo-4a applied to cell front, downward triangles = Dyngo-4a applied to cell rear) represent the mean from each experiment, which were used for statistical comparisons; error bars indicate mean ± standard deviation across experiments. Sample sizes: Control (n = 10), Dyngo-4a applied to cell front (n = 8), and Dyngo-4a applied to cell rear (n = 10). Statistical significance: * *p* < 0.05.

We lastly investigated how spatially controlled inhibition of endocytosis influences migration dynamics by quantifying speed and persistence in migrating cells treated with Dyngo-4a at either the front or rear compared to untreated controls. These analyses were restricted to cells that did not have another cell occluding the microchannels at either end, ensuring that the inhibitor exposure remains spatially restricted. Front-targeted Dyngo-4a treatment significantly increased migration speed relative to control (*p* = 0.047), whereas rear-targeted treatment produced a slight but non-significant reduction in migration speed relative to control (*p* = 0.409) (Figure 4A). Migration persistence was more variable across groups, with no significant differences detected (Control vs. front *p* = 0.142; Control vs. rear *p* = 0.4746) (Figure 4B). However, the wide variability observed in controls was reduced upon front-targeted inhibition (Control = 0.773 ± 0.150; Dyngo-4a front = 0.978 ± 0.011). These findings suggest that endocytosis at the leading edge of single cells may act as a regulatory brake on cell motility, while endocytosis at the trailing edge contributes to sustained forward cell movement.

Whether migrating cells completely occlude the Dyngo-4a gradients remains uncertain, as dextran imaging revealed variable outcomes: in some cases, gradients appeared to divert around the cell body, maintaining signal at both the front and rear, whereas in others, the gradient was fully blocked (Figure S7). These scenarios are not distinguishable by confocal or widefield fluorescence imaging alone, highlighting a limitation in interpreting gradient exposure during migration. Nonetheless, the ability of this platform to resolve distinct front- and rear-specific effects on cell migration dynamics demonstrates its value for dissecting spatial regulation of endocytosis in confined environments.

## 4. Discussion

Targeting the spatial regulation of endocytosis offers a new strategy to understand how cells navigate confined environments, particularly in the context of cancer cell invasion. Building on previous studies that implicate the role of endocytosis in adhesion turnover and migratory plasticity [9,30,31], we hypothesized that locally inhibiting CME would disrupt the polarized distribution of adhesion and endocytic machinery. Our findings support the hypothesis in part: front-targeted inhibition of endocytosis preserved or enhanced leading-edge enrichment of paxillin and AP-2, while rear-targeted inhibition abolished paxillin polarity and reduced AP-2 polarity. These outcomes suggest that endocytosis at the cell rear contributes to sustaining front–rear asymmetry of adhesion and endocytic complexes, whereas front-endocytosis is less essential for polarity maintenance [14,15,30,32,33,34].

However, the downstream effects on migration dynamics were not as strong as initially anticipated. Front-targeted inhibition produced a clear and significant increase in migration speed, consistent with the idea that CME at the leading edge may function as a brake on protrusion dynamics. By contrast, rear-targeted inhibition only slightly reduced migration speed and did not significantly alter persistence. If rear endocytosis is indeed critical for clearing adhesions and recycling membrane components, why were the effects on cell motility so modest? One possibility is that trailing-edge adhesion disassembly in confined channels can be compensated by other mechanisms, such as actomyosin contractility or physical shear from channel walls [5,6]. Alternatively, CME-independent pathways of endocytosis, such as caveolar uptake or micropinocytosis, may partially sustain recycling when dynamin-mediated CME is inhibited [8]. These compensatory mechanisms could blunt the impact of rear-targeted inhibition on whole-cell migration speed.

It is also important to consider whether our mechanistic model, that rear CME is uniquely critical for sustaining migration, is fully supported by the data. While rear-targeted inhibition disrupted polarity of adhesion markers, it did not strongly reduce migration speed or persistence. This raises the possibility that endocytic polarity and migratory output are not linearly coupled, or that polarity of AP-2 and paxillin is not the sole determinant of migration efficiency [9,31]. Instead, polarity may influence other cellular outcomes, such as signaling feedback loops, local turnover of receptors, or long-term migratory persistence over timescales beyond those measured here. Another possibility is that the observed differences in AP-2 and paxillin distributions reflect stalled structures resulting from Dyngo-4a treatment rather than an active loss of polarity [18,29], complicating interpretation. Dyngo-4a has also been reported to affect actin polymerization dynamics, which could influence adhesion remodeling independently of its role in dynamin inhibition [18,35]. Nevertheless, the asymmetric effects observed here, where front- and rear-localized Dyngo-4a treatments produced opposite changes in adhesion and endocytic polarity, are more consistent with localized inhibition of endocytosis rather than a global disruption of actin organization, although minor cytoskeletal contributions cannot be completely excluded.

Our findings also highlight sources of variability that may explain differences across cells. Dextran imaging showed that some cells fully occlude channels, while others allowed gradients to flow around the cell body. This heterogeneity could lead to differences in the degree of inhibitor exposure across cells, with some experiencing strongly polarized delivery and others a more diffuse distribution. Such variability may partially explain the moderate phenotypic changes in migration despite robust alterations in protein localization. Addressing this will require combining inhibitor delivery with live-cell imaging of endocytic and adhesion turnover in the same cells to directly link molecular changes with migratory outcomes. Live-cell imaging of AP-2 and paxillin during migration in this system will be especially informative, as prior work in SUM159 cells shows that focal adhesions and clathrin-containing adhesion complexes (a distinct class of adhesive structures associated with clathrin and endocytic machinery) coexist but follow different temporal patterns coordinated with actin architecture, thereby influencing cell-cycle progression and potentially migratory behavior [36].

Together, these results underscore the utility of our microfluidic system for probing localized molecular events in a physiologically relevant environment. By enabling targeted drug delivery to subcellular regions of migrating cells, our platform provides a valuable tool to dissect how adhesion and endocytic polarity shape migration under confinement. At the same time, our findings caution against overly simplistic mechanistic models. While endocytic polarity clearly shapes adhesion distributions, its effects on migration speed and persistence are more nuanced, likely buffered by redundant pathways and mechanical constraints. Future work will need to test whether specific endocytic cargoes, rather than global inhibition of CME, drive the migratory phenotypes we observed.

Additionally, validation across other cancer cell lines and in 3D ECM environments will help determine the generality of these mechanisms. Notably, the ECM of human tumors differs from that of normal tissues in matrix composition, pH, and cross-linking density [37], which collectively influence ECM physical properties such as stiffness and microarchitecture that mediate cell migration dynamics [38,39]. A key limitation of the current platform is that it is devoid of 3-D ECM. Future studies can incorporate 3-D matrices with tailored compositions, mechanical properties, molecular availability due to mass transport, and microarchitecture [40]. By integrating 3-D ECMs with defined cancer-relevant properties into microfluidic gradients and high-resolution microscopy used in the present study, researchers can identify critical cellular interactions through functional readouts and measurements at the cellular, sub-cellular, and molecular length scales [41].

More specifically, our results suggest that spatial control of trafficking may be a viable therapeutic target to disrupt single-cell tumor escape. However, the modest effects of rear inhibition on migration dynamics underscore the need to identify which specific trafficking pathways are indispensable for invasion, and which may be compensated by parallel mechanisms. This distinction will be crucial in designing strategies that selectively impair metastatic cells without broadly disrupting normal tissue homeostasis or immune function.

## 5. Conclusions

Our study shows that spatially restricted inhibition of CME alters adhesion polarity and migration behavior of breast cancer cells in confinement. Front-targeted inhibition preserved or enhanced leading-edge enrichment of paxillin and AP-2 and increased migration speed, while rear-targeted inhibition disrupted polarity without strongly reducing motility. These results suggest that CME makes distinct contributions at the front and rear of migrating cells, supporting the balance between adhesion turnover and protrusion dynamics. Heterogeneity in channel occlusion and gradient diversion, as revealed by dextran imaging, may underlie some of the variability in migration outcomes. Future studies will need to directly couple inhibitor delivery with live-cell tracking of adhesion and endocytic turnover, expand to multiple cell models, and assess cargo-specific internalization pathways. Together, these findings highlight endocytic polarity as an important regulator of confined migration and demonstrate the utility of spatial perturbation strategies for dissecting cancer cell behavior in microscale environments.

## Data Availability

All raw microscopy datasets shown in figures are publicly available on Zenodo at https://doi.org/10.5281/zenodo.17328946. The custom FIJI macro for front–rear cell division, along with an example segmentation pipeline and a representative raw image containing AP-2, paxillin, and nuclear channels for testing, is available at https://doi.org/10.5281/zenodo.17329261. All other data and analysis scripts are available upon reasonable request.

## Supplementary Materials

The following supporting information can be downloaded at: https://www.mdpi.com/article/10.3390/bioengineering12111148/s1.
