# Supplementary material for: Spatial Regulation of Endocytosis and Adhesion Formation Governs Breast Cancer Cell Migration Under Confinement
Source: Bioengineering (Basel). 2025 Oct 23;12(11):1148. doi: 10.3390/bioengineering12111148 (PMC12649363; doi:10.3390/bioengineering12111148)
Supplement: Supplementary file 1 [file bioengineering-12-01148-s001.zip › bioengineering-3937977-supplementary/Supplemental files/Supplemental figures/Supplemental figure captions.pdf]

Supplemental figures

Fig. S1

COMSOL modeling and validation of gradient formation in the microfluidic device.

(A) Simulated velocity field showing flow streamlines through the inlet, chambers, and outlet, with no flow in the microchannels.

(B) Location of the midplane slice used for the velocity magnitude simulation shown in (C).

(C) Z-profile slice through the migration channel array showing velocity magnitude across all 30 channels. Velocity approaches zero at the channel midplane, indicating a stable no-flow environment.

(D) Simulated validation of FITC-dextran gradient formation over time at three representative channel positions: (i) closest to the source inlet, (ii) center of the device, and (iii) farthest from the source inlet. Normalized concentration is plotted against distance from the FITC-dextran sink, showing progressive gradient development over 30 minutes post-injection.

Fig. S2

Structural characterization and optical calibration for quantitative gradient analysis in microfluidic assays.

(A) Scanning electron microscopy images of the microfluidic device showing key dimensions: chamber height (top left), channel height (middle left), channel width (bottom left), and an overview of the multiple-channel array (right). The middle and bottom left images, as well as the right image, were taken from wafer regions that were broken after metal coating, resulting in visible reflectance from the SU-8 sidewalls.

(B) Calibration curve of FITC-dextran fluorescence intensity (background subtracted) as a function of concentration. Data are fit with a linear regression with R^2 overlaid on the graph.

(C) 30-minute time-course of FITC-dextran gradient formation normalized across three representative channels: (i) closest to the source inlet, (ii) center of the device, and (iii) farthest from the source inlet (N = 3).

Fig. S3

Design and scale of the microfluidic gradient device used for cell migration assays.

(A) Image of the experimental setup showing a glass syringe connected to the microdevice through tubing and a stainless steel pin within a 35 mm dish.

(B) Top view of the fabricated device loaded with colored dyes to visualize the inlets and outlet. The device is shown next to a U.S. dime for scale. Inset: fluorescence image of 0.025 mg/ml FITC-dextran showing the bottom 15 channels, demonstrating uniform channel spacing (scale bar: 100 µm).

(C) Schematic of the microdevice illustrating the large inlet and outlet reservoirs relative to the narrow migration channels, which connect the gradient inlet (EGF) and cell seeding inlet. The reservoir-to-channel volume ratio enables stable gradient formation and sustained flow through the microchannels.

Fig. S4

Spatial distribution of AP2 and paxillin in migrating cells under EGF and Dyngo-4a treatment.

(A) Maximum intensity projection showing a top view of a portion of the device in an experiment where Dyngo-4a was applied to the same chamber as EGF (same image as shown in Error! Reference source not found.**C**). Cells endogenously express AP2-eGFP (green) and are immunostained for paxillin-mCherry (red) and Hoechst for the nucleus (blue). Tracked cells (6) are indicated by red numbers. Arrow indicates the direction of cell migration. Two representative regions are shown: (i) corresponds to panel (B); (ii) corresponds to panel (C). Scale bar: 50 μm.

(B) Cell in chamber with EGF and Dyngo-4a added. AP-2 and paxillin are broadly distributed across the cell. Scale bar: 10 μm.

(C) Cell in chamber with no EGF or Dyngo-4a added. AP-2 localizes to puncta concentrated in the midsection, and filopodia are visible at the periphery. Scale bar: 10 μm.

Fig. S5

Representative images of paxillin and AP-2 polarity under localized Dyngo-4a inhibition.

Spinning-disk confocal images of migrated SUM159 cells confined within microfluidic migration channels, fixed and stained for paxillin (red), AP-2 (green), and nuclei (blue). Cells migrate to the right, as indicated by the white arrow. Dashed microchannel boundaries removed for clarity. Scale bars: 5 μm.

(A) Control cells.

(B) Cells treated with Dyngo-4a at the front.

(C) Cells treated with Dyngo-4a at the rear.

Fig. S6

Stepwise image analysis workflow for quantifying front-rear localization of paxillin and AP-2.

(A) A maximum intensity projection of the background-subtracted paxillin fluorescence channel is generated in FIJI.

(B) Balloon segmentation is applied to define the cell boundary.

(C) The cell is divided into front and rear halves by iteratively adjusting the division until both regions have equal area using an automated script.

(D) The moments threshold is applied to isolate mature paxillin structures.

(E) Segmented paxillin signal displayed within the trailing and leading edge-ROIs.

(F) The same ROIs are applied to the AP-2 fluorescence channel for downstream front-rear quantification, which includes watershed analysis to separate overlapping AP-2 structures.

Fig. S7

Examples of cells that do not block versus fully block dextran gradients.

(A) Time-lapse fluorescence images of SUM-159 AP2-eGFP cells migrating in microchannels visualized with Alexa Fluor™ dextran, showing two representative cells (Cell 1 and Cell 2) over a 2-hour period. Yellow lines indicate regions of intensity measurement along channels. AP2-eGFP signal was used only for cell identification and not quantified in this analysis. Due to bleedthrough between green and red channels, AP2-eGFP and Alexa Fluor dextran signals could not be combined in the same panel. Scale bar: 50 µm.

(B) Gradient profiles for Cell 1 show continuous dextran signal at both the front and rear, indicating no blockage.

(C) Gradient profiles for Cell 2 show complete loss of dextran signal behind the cell body at all time points, consistent with full blockage of the gradient.
