# Supplementary material for: Spatial Regulation of Endocytosis and Adhesion Formation Governs Breast Cancer Cell Migration Under Confinement
Source: Bioengineering (Basel). 2025 Oct 23;12(11):1148. doi: 10.3390/bioengineering12111148 (PMC12649363; doi:10.3390/bioengineering12111148)
Supplement: Supplementary file 1 [file bioengineering-12-01148-s001.zip › bioengineering-3937977-supplementary/Supplemental files/Supplemental movies/Supplemental movie captions.docx]

# Supplemental movie captions

## Movie S1

COMSOL simulations were performed using a molecule with the same parameters as 10 kDa FITC-dextran (diffusion coefficient = 1.43 × 10^-10^ m^2^/s). The molecule was introduced into the top chamber of the device at a concentration of 0.1 mol/m^3^, with flow maintained through the outlet at 5 μL/h.

**Movie S2**

This movie shows the same cell featured in **Fig. 3A** and **Fig. S6A-F**, imaged across 12 z-planes at 0.5 μm intervals to capture the full cell volume within a microchannel (z-positions labeled in the top left). The 0 μm plane corresponds to the ventral membrane-glass interface, and the 4.5 μm plane represents the dorsal membrane in contact with the fibronectin-coated PDMS ceiling. The cell endogenously expresses AP2-eGFP (green) and is immunostained for paxillin-mCherry (red) and Hoechst for the nucleus (blue). Scale bar: 5 μm.

**Movie S3**

Rotation of a SUM-159 AP2-eGFP cell (Cell 2 of **Fig. 2C** and **Fig. 3A)** perpendicular to the migration axis, transitioning from ventral to lateral to dorsal views. The cell endogenously expresses AP2-eGFP (green) and is immunostained for paxillin-mCherry (red) and Hoechst for the nucleus (blue). Scale bar: 5 μm.

**Movie S4**

The full sequence of cell seeding, serum starvation, migration, and cell tracking in the microdevice, corresponding to the same experiment as displayed in **Fig. 2C-D**, **Fig. S4**. The timestamp is displayed in minutes. In this experiment, EGF and Dyngo-4a were added to the right chamber. Scale bar: 100 μm.
