# Supplementary material for: Spatial Regulation of Endocytosis and Adhesion Formation Governs Breast Cancer Cell Migration Under Confinement
Source: Bioengineering (Basel). 2025 Oct 23;12(11):1148. doi: 10.3390/bioengineering12111148 (PMC12649363; doi:10.3390/bioengineering12111148)
Supplement: Supplementary file 1 [file bioengineering-12-01148-s001.zip › bioengineering-3937977-supplementary/Supplemental files/Supplementary methods.pdf]

Supplementary Methods

S1. Design and fabrication of microfluidic migration devices

The micron-scale and multi-level features of the microfluidic migration device were first patterned onto silicon wafers using SU-8 photoresist (*1*). The fabricated device dimensions are designed as follows: 30 migration channels (5 μm tall, 20 μm wide, 150 μm long); 2 chambers (100 μm tall, 100 μm wide, 1400 μm long); 2 inlets and 1 outlet (radii 375 μm). Fabrication of multi-level features required two transparency photomasks designed in AutoCAD 2021: one defining the thicker chamber, inlets, and outlet regions at approximately 100 μm in height, and the other defining the thinner migration channels at approximately 5 μm in height. To create the master mold, a silicon wafer was spin-coated with negative SU-8 photoresist, where SU-8 2100 (Kayaku Advanced Materials, Inc., Y111075 0500L1GL) was used to form the thick features and SU-8 2005 (Kayaku Advanced Materials, Inc., Y111045 0500L1GL) was used for the thin channels. The wafer was exposed to UV light using a maskless aligner (Heidelberg Instruments, MLA 150) to ensure precise alignment of the two layers. After UV exposure, the SU-8 2100 layer was developed using SU-8 developer (Kayaku Advanced Materials, Inc., Y020100 4000L1PE), generating the negative relief of the design. The wafer underwent a hard bake at 150 °C for 2 hours to eliminate cracks and refine structural details. To prevent polydimethylsiloxane (PDMS) from adhering to the mold, silanization was performed by passivating the wafer with tridecafluoro-1,1,2,2-tetrahydrooctyl-1-trichlorosilane (United Chemicals Ltd, T2492) for 30 minutes in a fume hood.

The developed SU-8 master was then placed into a 150 mm petri dish (VWR, 10062-882) for PDMS replica molding (*1*). A 10:1 mixture of PDMS base elastomer to cross-linker (Dow Corning Corporation, Sylgard 184 Silicone Elastomer) was poured over the mold, degassed, and cured at 65 °C for 2 hours. The cured PDMS layer, approximately 0.55-0.65 cm thick, was peeled off and access ports were introduced by punching a 0.5 mm hole at the outlet using a

stainless steel biopsy punch (Robbins Instruments, RBP-05) and two 4 mm inlet holes using a biopsy punch (Integra LifeSciences Services, 33-34-P/25). These inlets were positioned symmetrically to ensure proper fluid distribution. The PDMS layer was irreversibly bonded to a No. 1.5 glass coverslip inside a 35 mm dish (MatTek, P35G-1.5-20-C) using plasma oxidation (30W, 650 mbar, 70 seconds; Harrick Plasma, PDC-001) followed by baking at 65 °C for 10 minutes. This multi-layer assembly process finalized the formation of the microfluidic migration chamber.

S2. Validation of dimensions of migration devices

Microstructural features of the SU-8 microfluidic migration device were characterized using scanning electron microscopy (SEM) at Ohio State University's Nanotech West Laboratory. A row of devices patterned on the wafer was fractured at specific positions to expose channel sidewalls for cross-sectional imaging. Prior to mounting, a thin layer of gold-palladium was deposited on the broken wafer pieces using a Cressington 108 Auto/SE Sputter Coater to reduce surface charging and enhance image contrast.

After sputter coating, samples were affixed to aluminum pin stubs using conductive carbon tape, and electrical grounding was further ensured using colloidal silver paste. SEM imaging was conducted using a Zeiss Ultra Plus field emission SEM. Samples were introduced via the loadlock chamber and mounted on a single stub mount holder. Images were acquired under high vacuum using the in-lens secondary electron detector at an accelerating voltage of 5.0 kV, a stage tilt of 45°, and a working distance of approximately 4.2-2.5 mm. Imaging was performed at 250-5000X magnification with a 60 µm aperture and a frame acquisition time of 15.9 seconds to capture the full cross-section of microchannels and assess their height, width, and sidewall integrity.

S3. Characterization of migration devices

Fluid dynamics and molecular transport within the migration device were modeled using COMSOL Multiphysics 6.2 (COMSOL Inc.), employing the CFD and Transport of Diluted Species modules. The device geometry was reconstructed based on experimental dimensions: migration channels measuring 5 µm (H) × 20 µm (W) × 150 µm (L), two chambers measuring 100 µm (H) × 100 µm (W) × 1400 µm (L), and inlets and outlets with radii of 375 µm and 250 µm, respectively. Default properties of water at 37°C were used to approximate phosphate-buffered saline (PBS).

A steady-state creeping flow model was implemented to simulate low-shear conditions relevant to cell migration. Boundary conditions included no-slip conditions along the walls, no-flux boundaries except at the two inlets and single outlet, and an outlet set to atmospheric pressure. A constant withdrawal rate of 5 µL/h was applied at the outlet, ensuring equal flow input from both inlets. To simulate molecular transport, a time-dependent diffusion model was solved for 10 kDa FITC-dextran, using a diffusion coefficient of 1.43×10^{-10} m^2/s calculated via the Stokes-Einstein equation (*2*). All domains were initialized at zero concentration, with boundary conditions set to 0.1 mol/m^3 in the source chamber and 0 mol/m^3 in the sink chamber. Simulations were run for 5 hours to analyze gradient formation and stability across the migration channels.

Fluorescence intensity profiles along the migration channels were extracted and normalized to assess gradient formation over time. The final steady-state concentration distributions were compared across channels at multiple, defined time points. Mesh refinement was performed to ensure numerical stability and minimize computational error.

Note: Although the diffusion coefficients of EGF (~6 kDa), Dyngo-4a (~0.34 kDa), and the 10 kDa FITC-dextran tracer differ in magnitude, these differences affect only the time required to reach equilibrium and not the steady-state concentration profile. Under the diffusion-dominated,

low-flow conditions of the microfluidic device, the gradient shape is determined by boundary concentrations and device geometry rather than absolute diffusivity.

S4. Gradient validation and fluorescence imaging setup

A chemotactic gradient in the migration device was characterized using 10 kDa FITC-dextran (Sigma-Aldrich, FD10S) and imaged with a spinning disk confocal microscope. After fabrication and bonding of the device to a No. 1.5 glass coverslip (Fisherbrand, 12544E), a stainless steel pin (New England Small Tube, NE-1310-03) was bent and connected to plastic tubing (0.020" ID x 0.060" OD Tygon® ND 100-80 Microbore Tubing, 56515), approximately 0.5 meters in length. The tubing was threaded through a drilled 0.5 cm diameter hole in a 35 mm plastic dish lid (MatTek, P35G-1.5-20-C) and connected to a 2.5 mL glass syringe (Hamilton Company, 81420) fitted with a metal needle hub (Hamilton Company, 7748-09). The syringe was mounted on a syringe pump (Harvard Apparatus, 70-3007) to control media withdrawal. Before attachment to the microdevice, the syringe, needle, tubing, and stainless steel pin were pre-filled with 1X PBS (Corning, 21-031-CV). The pin was connected to the device outlet, and the syringe pump was set to infuse at 5 µL/h to establish stable flow within the device.

After securing the device on the confocal microscope stage, 50-60 µL of 1X PBS (Thermo Fisher Scientific Inc., 10010023) was added to each inlet. To equilibrate pressures between the two inlets, the syringe pump was switched to withdrawal mode at 5 µL/h for 1.5 hours. Two pipettes were then used to simultaneously introduce equal volumes of 1X PBS and a concentrated solution of FITC-dextran into the respective inlets (less than 2 µL each). The FITC-dextran solution was prepared at a concentration that would yield a final inlet concentration of 1 mg/mL, accounting for an estimated 3.75 µL reduction per inlet volume due to prior flow. Following FITC-dextran introduction, a continuous withdrawal rate of 5 µL/h was maintained for up to 5 hours during imaging.

FITC-dextran fluorescence was captured using a 20× objective lens (NA 0.75, Nikon) and 488 nm excitation (300 ms exposure time, 25% power) at 37 °C. The Nikon Perfect Focus System was used to maintain focus at the glass-PDMS interface. Two adjacent fields of view were imaged to encompass all 30 migration channels.

In parallel, Alexa Fluor™ 647-conjugated dextran (Thermo Fisher Scientific Inc., D22914) was loaded into the device using the same procedure described above. Identical tubing, syringe, and pump settings were used, with the Alexa Fluor™ dextran introduced into one inlet while PBS was added to the opposite inlet. Cells were introduced into the device, allowed to spread, and serum-starved. Following the serum starvation period, Alexa Fluor™ dextran was introduced along with EGF into the inlets, and imaging was performed on the spinning disk confocal system with 640 nm excitation (300 ms exposure time, 30% power) every 10 minutes for 2 hours. Fluorescence was monitored every 30 minutes for up to 2 hours to assess gradient stability and cell interactions with the channel walls. Note: Cells exhibited cytoxicity upon addition of Alexa Fluor™ dextran, which limited its use for long-term migration assays.

Images were analyzed in using the following workflow: (1) Due to the high concentration of FITC-dextran and Alexa Fluor™ dextran (1 mg/mL) used for fluorescence detection, out-of-plane fluorescence from the chamber regions resulted in saturation along the channel edges, typically spanning 15-20 μm in length. Therefore, fluorescence intensity profiles were measured exclusively in unsaturated regions within each channel. (2) For each device, the intensity profiles of the channel closest to the source inlets, channel in the center of the device, and channel farthest from the source inlets were measured and compared over time. (3) Each intensity profile was background-subtracted and normalized to the maximum intensity within the channel region in each frame, excluding saturated areas. The gradient profile was analyzed every 1 minute up to 30 minutes after FITC-dextran introduction, then every 30 minutes for up to 5 hours.

S5. Cell loading and equilibration in migration devices

After bonding the microfluidic device to a No. 1.5 glass coverslip in a 35 mm dish (see **Gradient validation and fluorescence imaging setup**), the device was treated with high-intensity UV light for 30 minutes. A UV-sterilized syringe, needle, tubing, and stainless steel pin filled with 1X PBS were then connected to the device outlet. The device was first wetted with PBS by manual infusion of PBS into the device. PBS was then added to the device inlets and the syringe was manually withdrawn to remove any created bubbles in the device. Excess PBS was removed from the inlets and replaced with 50-60 uL of 100 μg/mL fibronectin (Sigma-Aldrich, FC010), depending on the thickness of the PDMS. The fibronectin was manually withdrawn to fill the device, needle, and some of the tubing to account for possible backflow of media back into the device during the fibronectin incubation period. The preceding method was used to exchange old media in the device, and is adopted for the rest of the procedures written in the following sections. After fibronectin was introduced, 1 mL of PBS was added to the dish surrounding the device to minimize evaporation of media inside the channels during long-term culture and imaging. The device was treated with fibronectin for 1.5 hours at 37 °C for the PDMS to absorb the fibronectin and become conducive for cell attachment and growth.

Cells were harvested from culture plates using 0.05% Trypsin/EDTA (Thermo Fisher Scientific Inc., 25200056) and centrifuged at 1500 rpm for 5 minutes. The cell pellet was resuspended in phenol red-free F-12 complete media at a concentration of approximately 1×10^5 cells/mL. The device was washed with PBS before being filled with F-12. Excess media was removed from one of the inlets and replaced with the cell suspension. A slightly larger volume of the cell suspension was added to this inlet compared to the opposite inlet to drive cell movement into both chambers. The suspension was allowed to settle for 5 minutes before repeating this process 3-5 times until an adequate number of cells accumulated in the chamber closest to the

inlet. The device was placed in a 37 °C incubator, and cell spreading was monitored every 10 minutes using a dual phase contrast/fluorescence LED microscope (Leica, PAULA).

After cell spreading, the device was taken out of the incubator and the media in the inlets were exchanged with equal volumes of serum-free phenol red-free F-12 (50-60 μL, depending on the thickness of PDMS) to induce cell cycle synchronization and heighten the cell response to growth factors (*20*). The device was then returned to the incubator and connected to a syringe pump (Harvard Apparatus, 70-3007) for 1.5 hours at a withdrawal rate of 5 μL/h. This stabilization period was imaged every 10 minutes using the Leica PAULA microscope.

S6. Time-lapse imaging and treatment setup for migration assays

Following serum starvation, time-lapse imaging of cell migration under different conditions was performed using a dual phase contrast/fluorescence LED microscope (Leica, PAULA). To introduce EGF and/or endocytic inhibitors into the microfluidic device, the lid of the 35 mm dish was briefly removed and replaced after addition.

Cell migration was first imaged in the presence of an EGF gradient. After serum starvation, two pipettes were used to simultaneously introduce equal volumes of serum-free F-12 and a concentrated EGF (Thermo Fisher Scientific Inc., AF-100-15-500UG) solution into each inlet of the device (5-6 μL, depending on PDMS thickness). The EGF stock solution was prepared at a concentration that would result in a final inlet concentration of 20 ng/mL after spiking, accounting for an estimated 3.75 μL reduction per inlet volume due to prior flow. For consistency, EGF was always introduced in the inlet opposite the chamber where cells had been seeded.

Next, cell migration was imaged in the presence of an EGF gradient with 5 μM Dyngo-4a (Abcam, ab120689). After adding EGF to one inlet, two additional pipettes were used to simultaneously introduce equal volumes of serum-free F-12 and a concentrated Dyngo-4a solution into either the same inlet as EGF or the opposite inlet (<1 μL, depending on PDMS

thickness). Following media addition, the dish lid was replaced, and cells were imaged every 10 minutes for up to 5 hours using the Leica PAULA system.

References

1. D. Qin, Y. Xia, G. M. Whitesides, Soft lithography for micro- and nanoscale patterning. *Nat Protoc* **5**, 491–502 (2010).

2. H. Wen, J. Hao, S. K. Li, Characterization of Human Sclera Barrier Properties for Transscleral Delivery of Bevacizumab and Ranibizumab. *J Pharm Sci* **102**, 892–903 (2013).
