# Supplementary figures and images for: Spatial Regulation of Endocytosis and Adhesion Formation Governs Breast Cancer Cell Migration Under Confinement
Source: Bioengineering (Basel). 2025 Oct 23;12(11):1148. doi: 10.3390/bioengineering12111148 (PMC12649363; doi:10.3390/bioengineering12111148)

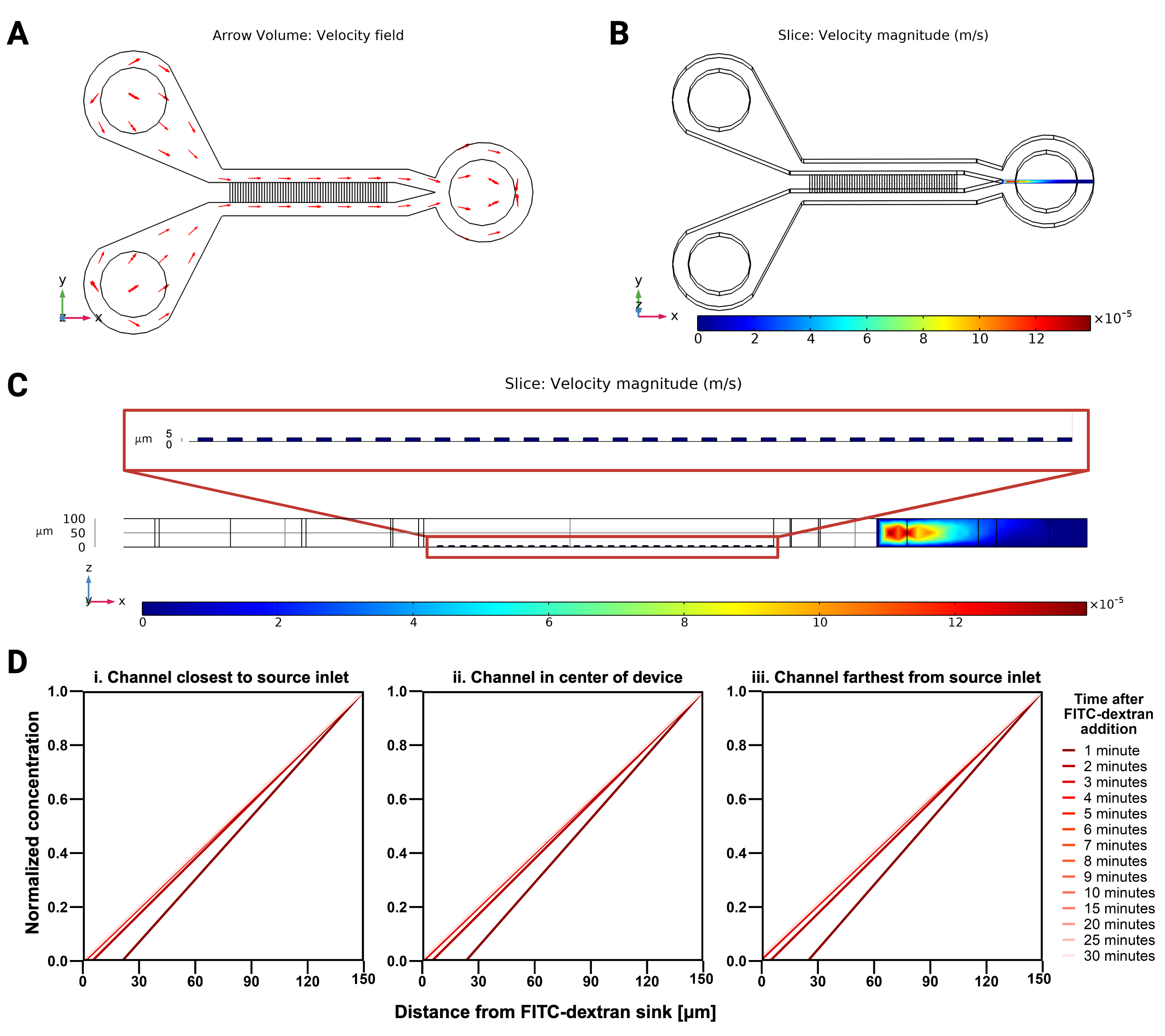

Supplement: Supplementary file 1 [file bioengineering-12-01148-s001.zip › bioengineering-3937977-supplementary/Supplemental files/Supplemental figures/Fig S1.png]

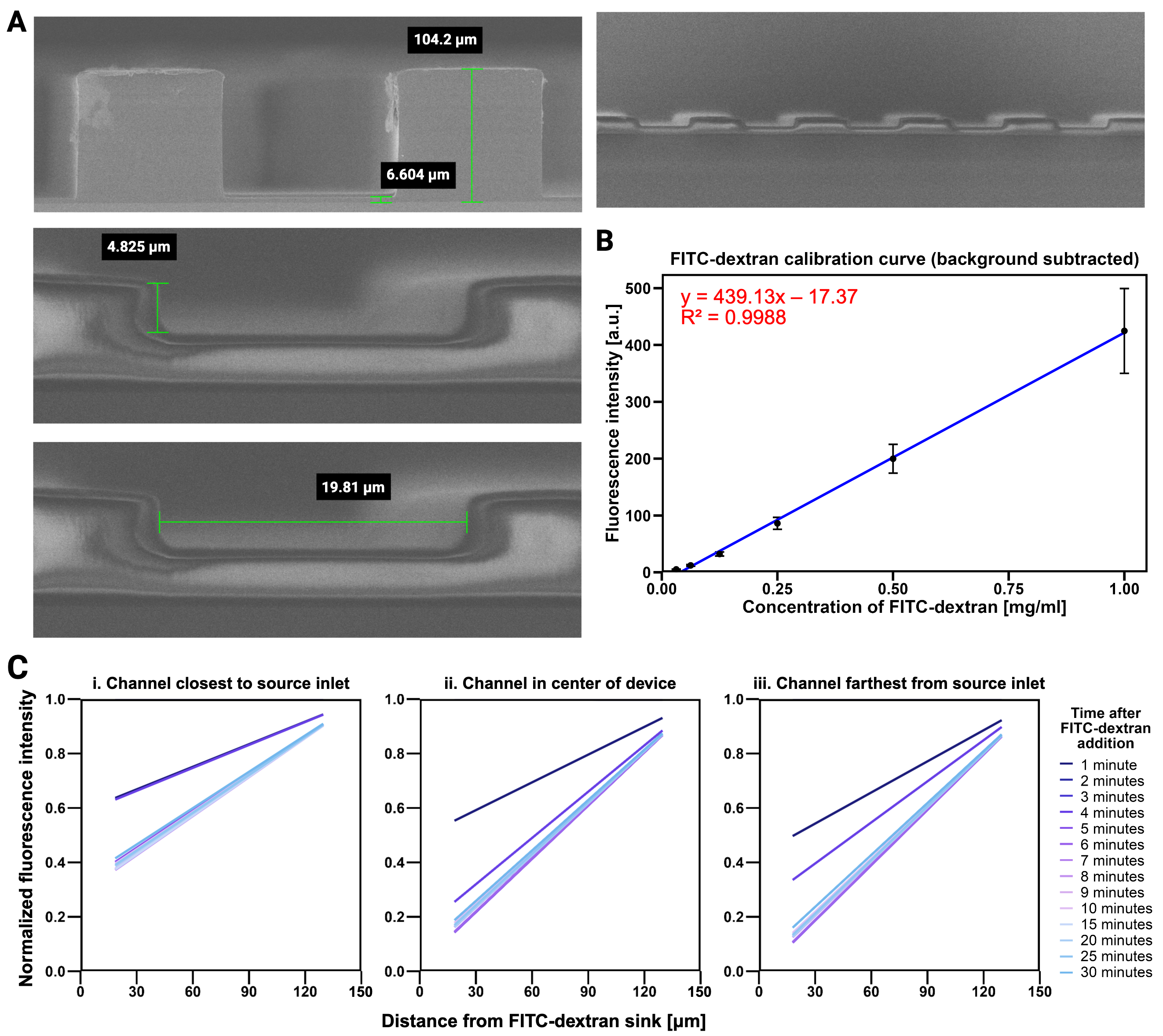

Supplement: Supplementary file 1 [file bioengineering-12-01148-s001.zip › bioengineering-3937977-supplementary/Supplemental files/Supplemental figures/Fig S2.png]

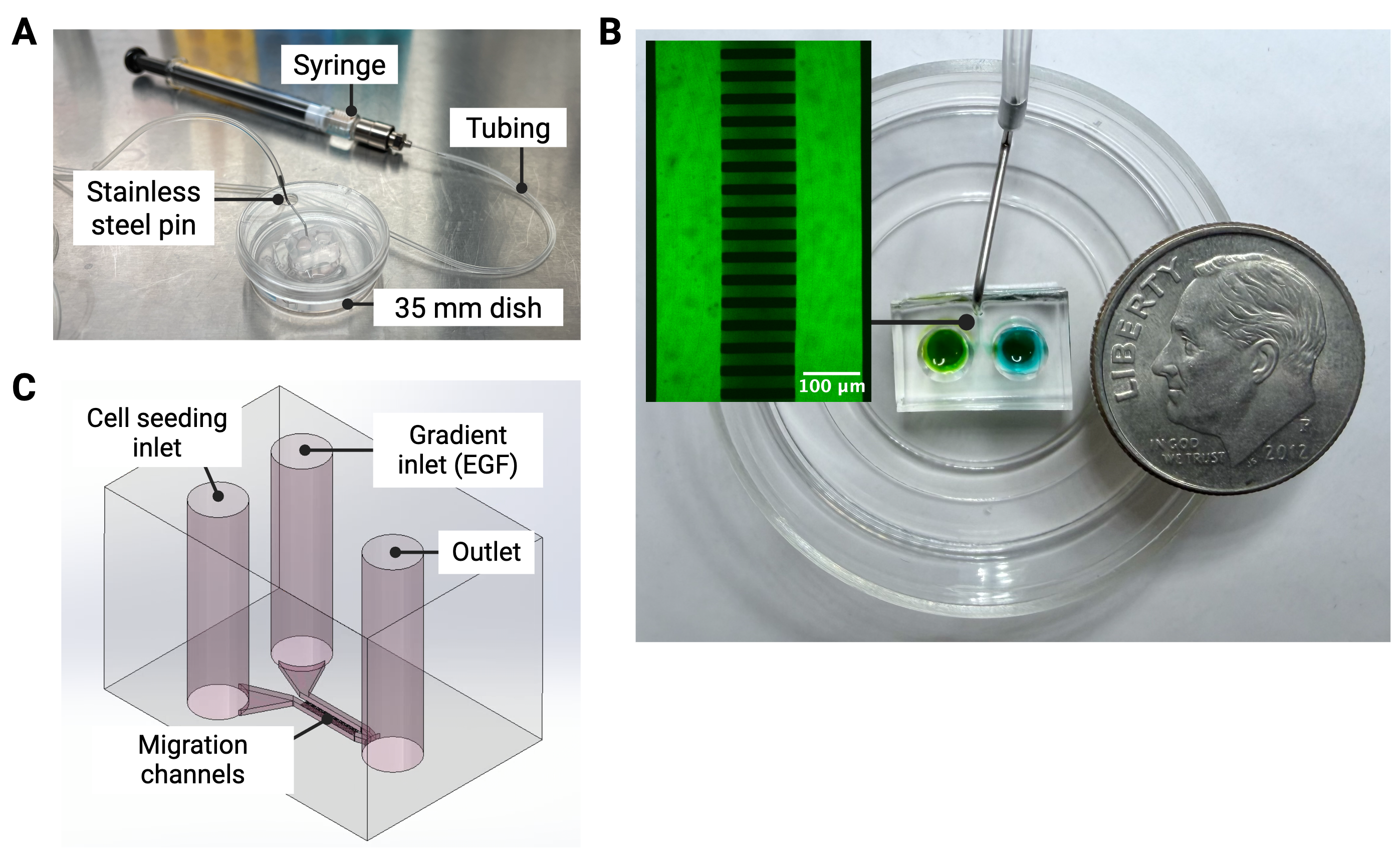

Supplement: Supplementary file 1 [file bioengineering-12-01148-s001.zip › bioengineering-3937977-supplementary/Supplemental files/Supplemental figures/Fig S3.png]

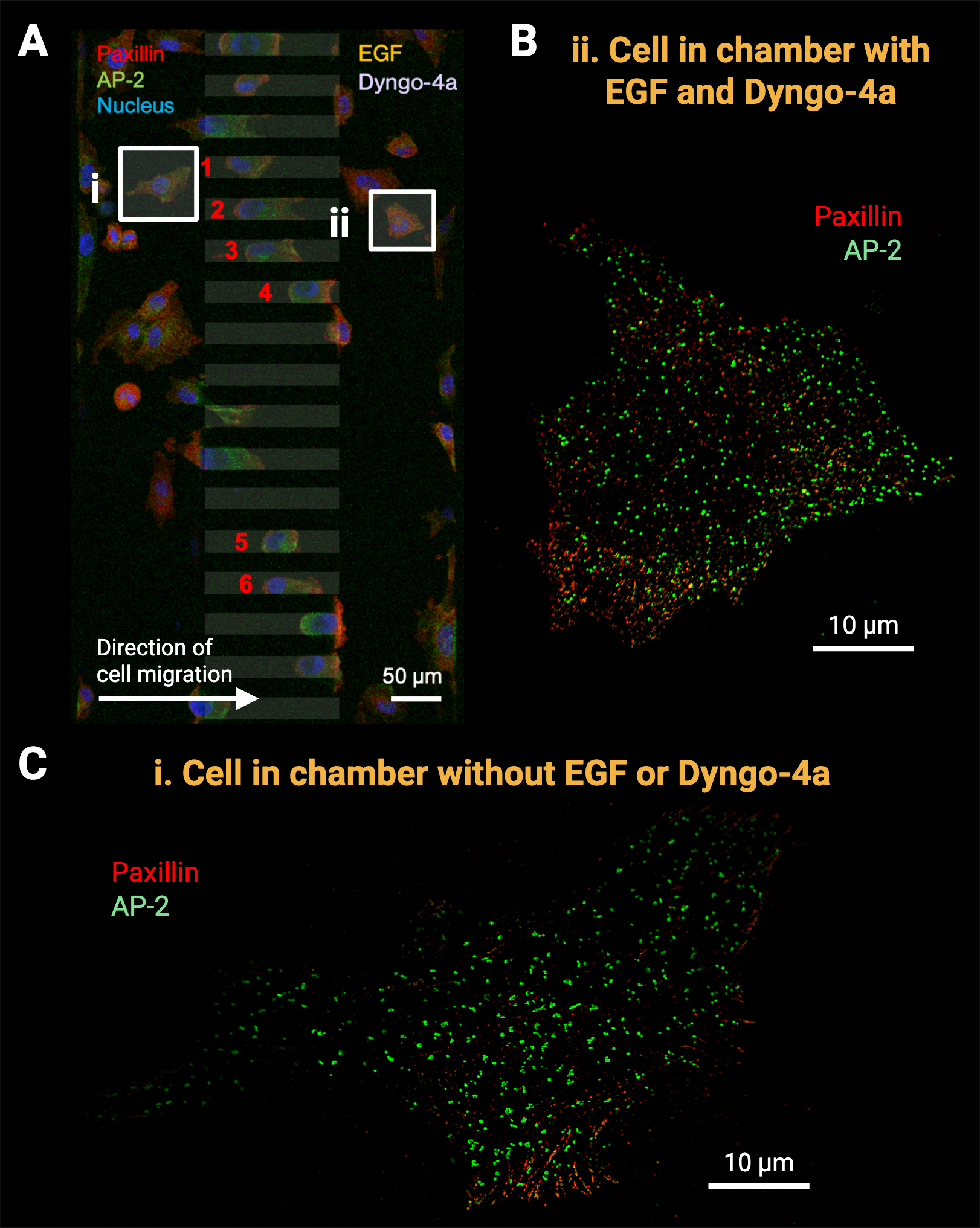

Supplement: Supplementary file 1 [file bioengineering-12-01148-s001.zip › bioengineering-3937977-supplementary/Supplemental files/Supplemental figures/Fig S4.png]

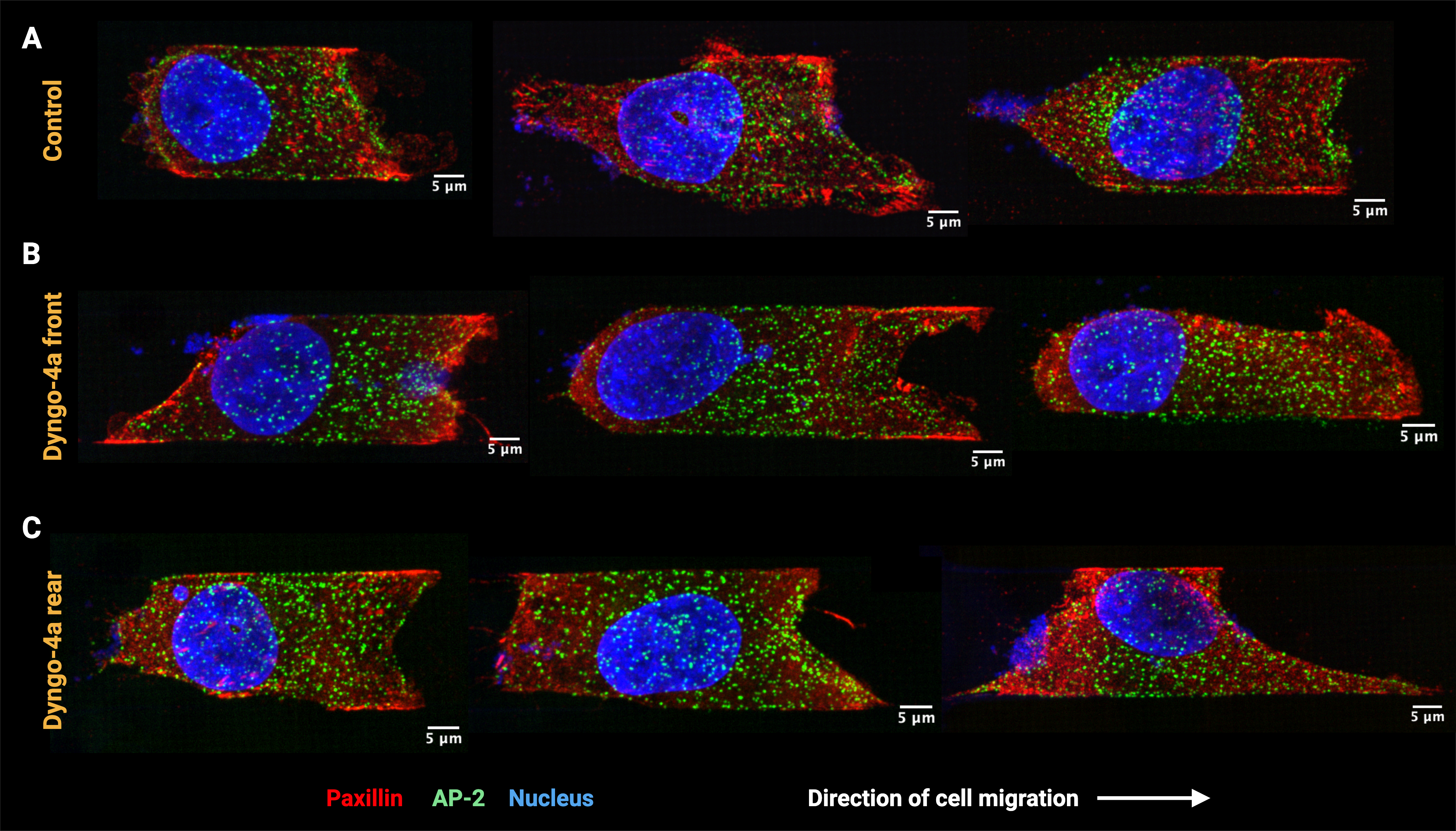

Supplement: Supplementary file 1 [file bioengineering-12-01148-s001.zip › bioengineering-3937977-supplementary/Supplemental files/Supplemental figures/Fig S5.png]

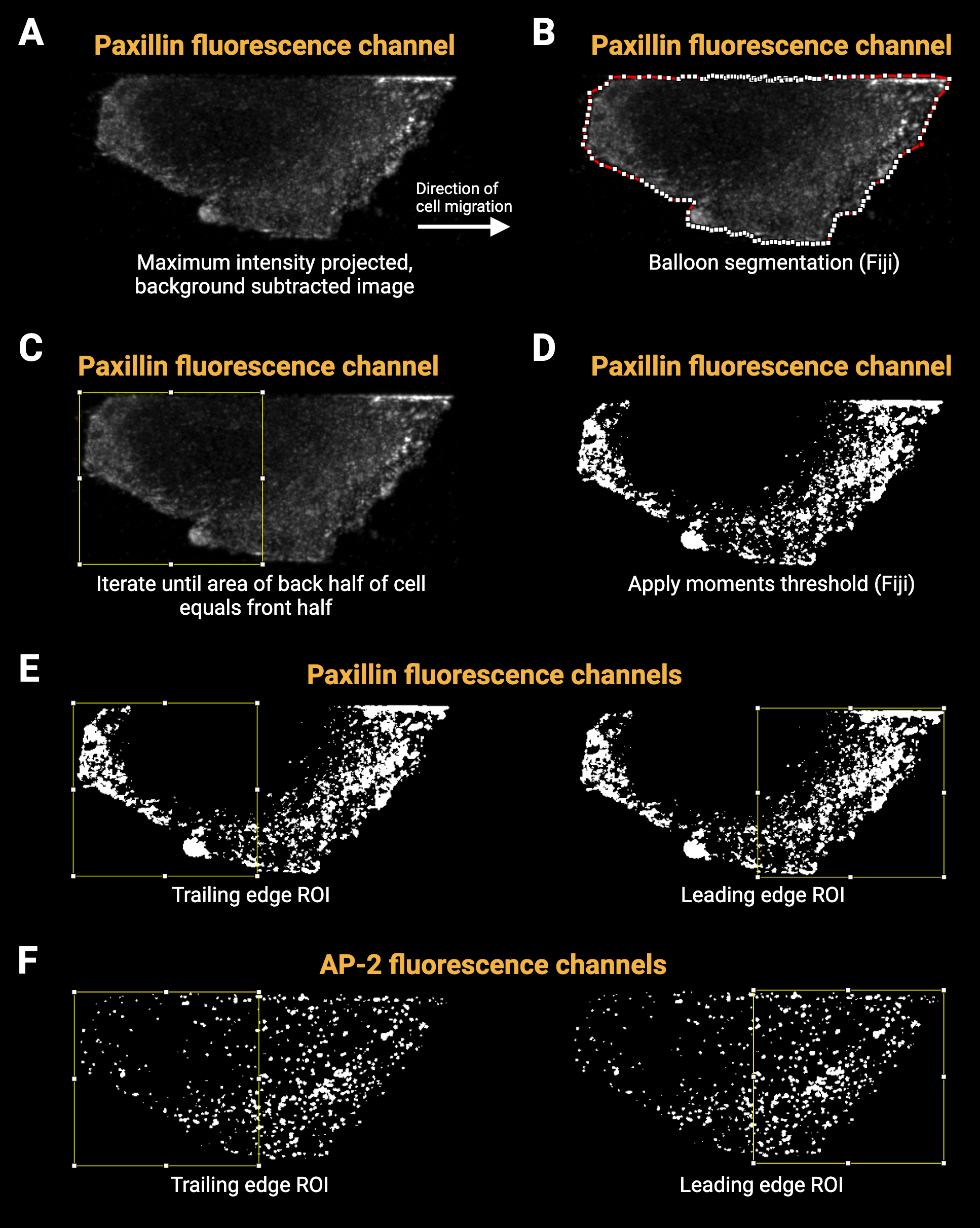

Supplement: Supplementary file 1 [file bioengineering-12-01148-s001.zip › bioengineering-3937977-supplementary/Supplemental files/Supplemental figures/Fig S6.png]

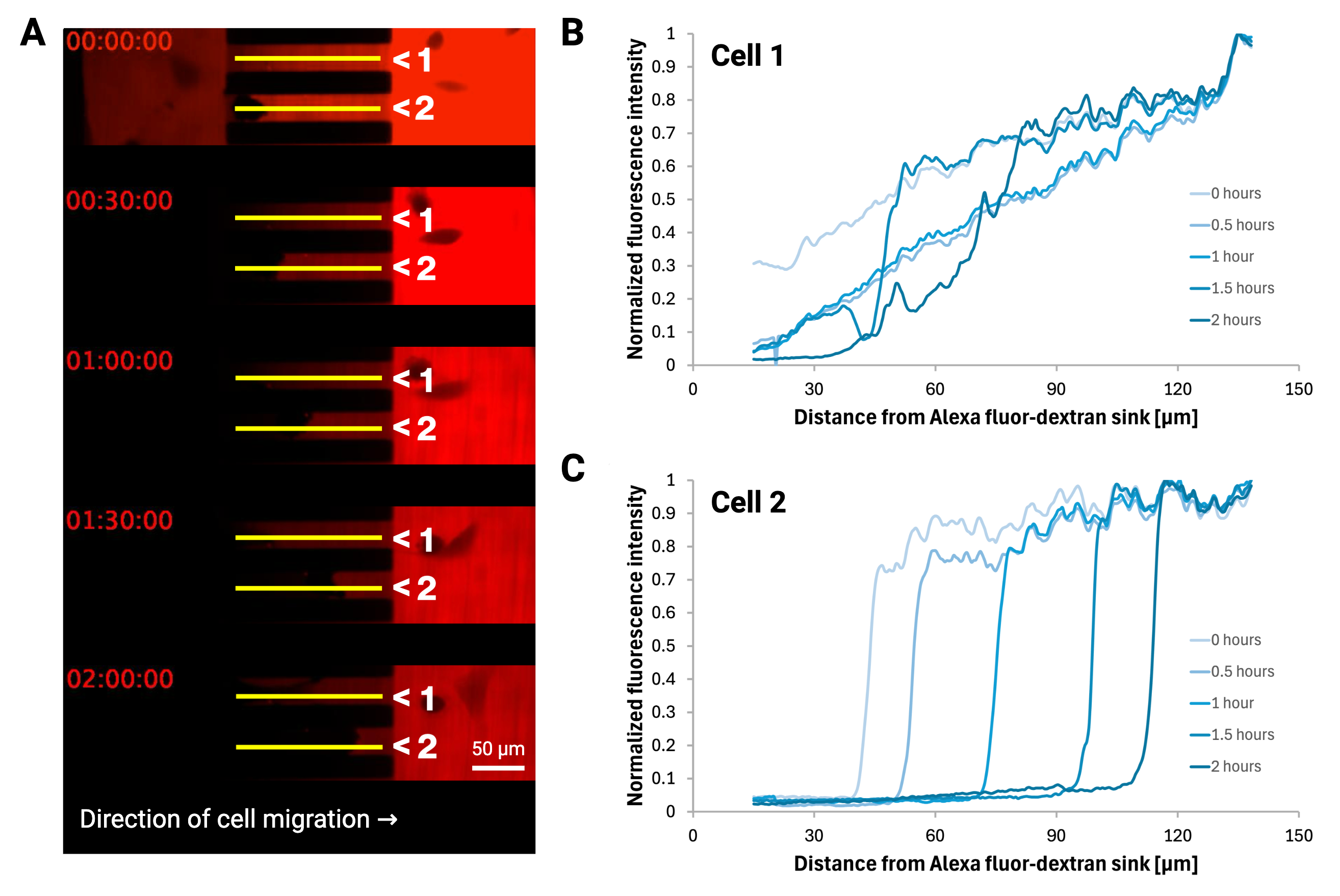

Supplement: Supplementary file 1 [file bioengineering-12-01148-s001.zip › bioengineering-3937977-supplementary/Supplemental files/Supplemental figures/Fig S7.png]
